# Proteins in Relation to Vigor and Viability of White Lupin (*Lupinus albus* L.) Seed Stored for 26 Years

**DOI:** 10.3389/fpls.2017.01392

**Published:** 2017-08-11

**Authors:** Malwina Dobiesz, Agnieszka I. Piotrowicz-Cieślak

**Affiliations:** Department of Plant Physiology, Genetics and Biotechnology, Faculty of Biology and Biotechnology, University of Warmia and Mazury in Olsztyn Olsztyn, Poland

**Keywords:** lupin seeds, storability, protein, conglutins, long term storage seeds

## Abstract

The aim of the study was to evaluate the vigor and viability as well as to determine and compare the contents of selected protein fractions of white lupin (*Lupinus albus* L.) seeds stored for 26 years at temperatures of -14°C and +20°C. The seeds stored at -14°C germinated in 86.3%, while the seeds stored at +20°C did not germinate at all. The viability evaluation was confirmed by the measuring electroconductivity of seed exudates. In seeds stored at -14°C the contents of γ, δ, and β conglutin were 14, 4 and 69 mg g^-1^ fresh mass, respectively, while in seed stored at +20°C they were 15.5, 3, 65 mg g^-1^ fresh mass, respectively. One-dimensional electrophoresis of γ and δ conglutin fractions indicated the presence of several intense polypeptide bands with molecular weights from 23.0 to 10.3 kDa. Polypeptide bands with a molecular weight of 22.4 and 19.8 kDa exhibited almost two times higher expression in the seeds stored at -14°C compared to the seeds stored at +20°C. Electrophoresis revealed 310 protein spots on the maps generated for seeds stored at -14°C, and 228 spots for seeds stored at +20°C. In seeds stored at +20°C most polypeptide subunits had a pI ranging from 4.5 to 7 and a molecular weight of 10–97 kDa. The greatest differences in the contents of polypeptides between the analyzed variants was observed within the range of 20–45 kDa (-14°C: 175, +20°C: 115 protein spots) and within the range of 65–97 kDa (-14°C: 103, +20°C: 75 protein spots). In seeds stored at +20°C, a clear decline in basic (8–10 pI) polypeptides was observed. The study demonstrated that the polypeptides identified as γ and δ conglutins are probably closely related to vigor and viability of seeds.

## Introduction

Legumes and cereals are the main sources of proteins in human diets. Legumes are important foods for people with specific nutritional preferences, like vegans, vegetarians, and hyperlipidemia patients, who must observe lipid restriction (Arozarena et al., 2001). In addition to the nutritive value, legume seed proteins affect food structure and stability (Makri et al., 2005). First of all, however, they are vital components of seeds, affecting their vigor and potential for producing healthy seedlings. The germplasm of the most promising varieties is deposited in gene banks and subjected to long term storage. With the passage of time, adverse changes in seed macromolecules and intracellular systems accumulate and eventually they lead to the loss of seed viability. Diverse biochemical and physiological disturbances accumulate in seed and the rate of this deterioration is determined by the storage temperature and relative humidity (RH) (Priestley, 1986; Ratajczak et al., 2015). There are several hypotheses about the mechanics of seed aging. The main one has been proposed by Harman (1992). He assumed that the basic source of aging, in various organisms, not only plant seeds, is the accumulation of cell damage caused by reactive oxygen species (ROS) (Harman, 1992; Ratajczak et al., 2015). The accumulation of ROS in seeds results in damage to DNA, lipid, and proteins. Severe protein damage caused by oxidative stress may lead to a reduction in seed vigor and viability (Ratajczak et al., 2015; Barreto and Garcia, 2017).

Proteomic analysis of seeds should thus provide an insight into the effects of storage conditions on seed quality and longevity. Analysis of proteins extracted from the seeds of legume family is rather daunting. The main limitations of proteomic analysis are associated with the great diversity of proteins in terms of physico-chemical properties and significant differences in their amounts (Martínez-Maqueda et al., 2013). The vast majority of the isolated proteins are storage proteins. Proteins playing metabolic, structural, regulatory or defensive roles are represented less frequently and are often masked by storage proteins (Magni et al., 2007).

Analysis of the seed proteome may be more efficient when modern methods for fractioning and purification are applied (Berghout et al., 2015). These methods are based on cell fracture followed by protein solubilisation and/or precipitation in order to obtain the desired protein fraction. Of paramount significance is the minimisation of the effects of other agents (e.g., phenolic compounds) on the electrophoresis result (Martínez-Maqueda et al., 2013). The fractioning process requires that large quantities of water, energy and reagents be used. The disadvantages of this method also include significant losses of material, particularly during degreasing and drying (Berghout et al., 2015).

In the seeds of lupins, the main storage proteins are conglutins which, based on the electrophoretic mobility, were divided into four families, i.e., α, β, γ, and δ conglutins. δ Conglutin content of white lupin mature seeds was estimated to a level of 10–12%; however, the latest studies suggest a lower content of approximately 3–4% (Duranti et al., 2008).

This discrepancy most probably results from inaccurate purification of fractions during earlier studies. δ Conglutin is a monomer with a low molecular weight (Salmanowicz and Przybylska, 1994), comprising two subunits with weights of 4 and 9 kDa (Duranti et al., 2008). δ Conglutins are water soluble and include enzymatic proteins and inhibitors (Klupšaitė and Juodeikienė, 2015). Therefore, in a 2D electrophoretic image, they are often masked by the more abundant proteins (mainly γ conglutins). β Conglutin found in cotyledons accounts for 44–45% of all storage proteins, and is thus the most abundant protein present in the seeds of all lupin species. This protein is a tetramer whose monomers are comprised of polypeptides with a molecular weight of 16–70 kDa (Duranti et al., 2008), resulting in a complex pattern in a 2D electrophoretic image. γ Conglutin is a protein specific for lupin seeds and accounts for approximately 4–5% of the total protein of mature seeds. γ Conglutin is a hexamer whose every monomer is composed of two heterogeneous subunits with molecular weights of 17 and 29 kDa. However, the heterogeneity of γ conglutin subunits is not as clear as it is in the case of β conglutin subunits (Duranti et al., 2008). β Conglutin and γ conglutin are characterized by minimum solubility at the pH value of four to five. The manipulation of protein solubility, and the application of appropriate techniques using hydrodynamic properties of proteins, may help obtain the desired protein fractions (Klupšaitė and Juodeikienė, 2015; Muranyi et al., 2016). However, it is not always possible to obtain a uniform fraction, which is due to the fact that many proteins exhibit intermediate solubility (Nadal et al., 2011). γ Conglutin at neutral pH is readily soluble in water and in salt solutions (Duranti et al., 2008).

Decreased contents of soluble carbohydrates, biogenic amines and proteins was detected in yellow lupin (*Lupinus luteus* L.) seeds subjected to 29 years storage at room temperature (Dobiesz et al., 2017). The physiological role of the proteins correlating with seed vigor is pretty straightforward in some cases, for instance in free radical scavenging proteins. However, it was demonstrated that seed storability can be strongly affected by conglutins too. These proteins are considered storage molecules, and should undergo proteolysis at the onset of seed germination. It was interesting, therefore, to find out if the significance of conglutins in determining seed vigor and viability is a characteristic of most members of this protein group or just a few specific molecules. The objective of this paper was to fractionate white lupin seed conglutins into β, γ, and δ to obtain possibly precise 2-D electrophoretic separations and verify the hypothesis that most or some of the identified proteins could be considered as key molecules affecting seed vigor and vitality.

## Materials and Methods

The study material comprised seeds of white lupin (*L. albus* L.), Hetman variety. Seeds were stored in linen sacks, inside hermetically sealed glass ‘twist’ jars at -14°C and 50% (RH) and under laboratory conditions [temperature about +20°C and 44% (RH) for 26 years]. Hair hygrometer [model TR415 (C.P. Poland)] was used to measure the air RH. At the beginning of the experiment (in 1989) seeds contained 8.8% of water. The water content of the seeds after 26 years of storage (in 2015) was found to be 7.0% (storage at -14°C) and 4.8% (storage at +20°C). Water content was determined with the gravimetric method and expressed in relation to seed dry-weight (5 g samples were used for each replication of these determinations).

The vigor and viability of white lupin seeds (International Seed Testing Association [ISTA], 1999) were measured. Seed germination was carried out in an incubator with a constant temperature of +20°C. One hundred seeds of white lupin were placed evenly on a moist germination paper. The germination results were recorded as a percent of seed with embryonic roots emerging within 7 days of incubation. Subsequently, the length of stems and roots was measured and the fresh and dry weight of the seedlings was determined. In order to determine the electrical conductivity of seed leachates, the seed surfaces were sterilized by immersing them in 1% sodium hypochlorite for 2 min; the seeds were then washed in distilled water for 3–4 min. Subsequently, a seed sample (100 seeds) was soaked in milliQ water (250 ml) at 20°C for 24 h. The electroconductivity of seed leachate was measured with a conductometer (model HI 2315, Hanna Instrument). The measurements were conducted in triplicates for each batch of seeds. The electroconductivity was related to seed fresh weight and expressed in μS cm^-1^ g^-1^.

### Isolation of Proteins

Protein isolation was carried out following the method of Rubio et al. (1998) with minor modifications. The flour defatted with chloroform/methanol (2:1 v/v) was subjected to extraction with 0.2 M phosphate buffer (pH 8) containing 0.5 M NaCl, and the extract was sonicated (30 min at 4°C) and centrifuged (12,000 *g*, 30 min, 4°C). The supernatant pH was adjusted to pH 4.5 with acetic acid, it was then shaken (30 min) and centrifuged (28,000 *g*, 30 min, 4°C). The pellet was dissolved in borate buffer (pH 8), and then it was brought to 4.5 and centrifuged (28,000 *g*, 30 min, 4°C). The conglutin β precipitate, thus obtained, was dissolved and dialysed against a large amount of deionised water (48 h, 4°C) and then it was lyophilised. Next 608 g L^-1^ (NH_4_)2SO_4_ was added to the supernatant in order to isolate γ and δ conglutins. The solution was shaken for 2 h at 4°C and centrifuged (28,000 *g*, 30 min, 4°C). The pellet was dissolved and subjected to dialysis against a large amount of distilled water (48 h, 4°C). Next the solution was lyophilised. Protein determination was carried out according to Bradford (1976).

### 1D-Electrophoresis

The analyzed protein fraction was dissolved in a buffer containing: 0,0625 M Tris/HCl (pH 6.8), 2% SDS, 10% glycerol and 5% 2-mercaptoethanol, so as to obtain the final protein concentration of 2 mg ml^-1^. The samples were incubated in a water bath for 5 min. at 100°C. Next they were cooled down and loaded to a 10% polyacrylamide gel (7.0 × 10.0 cm) and subjected to SDS-PAGE electrophoresis (Mini PROTEAN Tetra System; Bio-Rad) at 200 V for 40 min. After protein separation the gels were stained in colloidal Coomassie Brilliant Blue G-250 (Sigma). Gel images were digitized with Gel Doc EZ Imager (Bio-Rad). Electrophorograms were analyzed using ImageLab software (Bio-Rad).

### Two-Dimensional Gel Electrophoresis

Rehydration and isoelectric focusing was carried out with a PROTEAN IEF Cell (Bio-Rad). The isolated proteins (70 μg of protein) were dissolved in a rehydration buffer of the ReadyPrep^TM^ 2-D Starter Kit (Bio-Rad) and added into a well of the focusing plate into which an IPG strip (ReadyStrip^TM^ 7 cm, pH 3-10, Bio-Rad) was then inserted. Twelve-hour passive rehydration was carried out. After rehydration was completed, isoelectric focusing was carried out (conditions: preparatory stage (250 V/15 min), voltage increase stage (250–4000 V/2 h), isoelectric focusing (rapidly increasing voltage from 4000 to 20000 V). After isoelectric focusing was completed, IPG strips were equalized in buffer I (6 M urea, 2% SDS, 0.375 M Tris-HCl with pH 8.8, 20% glycerol, 130 mM DTT; Bio-Rad) and buffer II (6 M urea, 2% SDS, 0.375 M Tris-HCl with pH 8.8, 20% glycerol, 135 mM iodoacetamide; Bio-Rad) for 10 min. The second stage involved separation of proteins in 12.5% polyacrylamide gels (7.0 cm × 10.0 cm) and in the case of β conglutin additional separation was carried out in a 10% polyacrylamide gel (7.0 cm × 10.0 cm), using the Mini PROTEAN Tetra System electrophoresis apparatus (Bio-Rad). Electrophoresis was carried out for 50 min in alternating current: 90 V (15 min) and 200 V. After the electrophoresis was completed, gels were dyed with a colloidal solution of Coomassie Brilliant Blue G-250 (Sigma). The gels were visualized with a Gel Doc EZ Imager scanner (Bio-Rad). The protein maps were analyzed in the PDQuest^TM^ Basic program (2-D Gel Analysis Software; Bio-Rad).

### Identification of Proteins

Selected protein spots were excised from the gel and crushed. The destaining was carried out in 50% acetonitrile (ACN) solution containing 50 mM NH_4_HCO_3_. After destaining the gel fragments were dehydrated with 100% ACN. They were then reduced by placing in NH_4_HCO_3_ solution (10 mM) containing DTT (100 mM) and incubated for 30 min at 57°C. Then they were dehydrated again with 100% ACN. In order to obtain cystein alkylation the gels were transferred for 45 min to darkness and incubated in iodoacetamide [50 mM in NH_4_HCO_3_ (100 mM)]. Next the gels were washed in NH_4_HCO_3_ (100 mM) and dehydrated in 100% ACN. Trypsin digestion (10 ng μl^-1^ w 25 mM NH_4_HCO_3_) was carried out overnight at 37°C. The resulting peptides were extracted with a solution of TFA (0.1%) and acetonitrile (2%). The peptide separation and determination of peptide masses was carried out using the Orbitrap spectrometer (Thermo). The measured peptide masses were compared to the records from the Viridiplantae database using the MASCOT software.

### Statistical Analyses

All biochemical analyses were carried out in at least three replicates, whereas the morphological observations were carried out in 10 replicates. The results are given as a means ± SD. *t*-Student test was used when comparing pairwise averages of independent samples for all parameters recorded at different storage temperatures, provided their distribution was normal and variances were uniform. In the cases when the distribution of a parameter values deviated from normal or variances were not uniform, the Cochran-Cox test was used.

## Results

### Seeds Viability

The seeds stored at -14°C germinated in 86.3%, while the seeds stored at +20°C did not germinate at all (**Table 1**). The vigor of seedlings was determined on the basis of the average root length and stem length. The average root and stem length of seedlings grown from seeds stored at -14°C was 1265 and 449 mm, respectively. Seed leachate electroconductivity of seeds stored at +20°C was twice as high as of seeds stored at -14°C (**Table 1**).

**Table 1:** White lupin germination (%), root and stem length (mm), seedlings fresh (mg) and dry (%) mass, water content (%) and electroconductivity (mS g^-1^ dry mass), in seeds stored for 26 years at different temperatures (data are expressed as the mean of 10–15 independent repeats ± SD, means in each column followed by the same letter are not significantly different *p* ≤ 0.01).

	Storage temperature	
	-14°C	+20°C
Germination, %	86.3 ± 2.06	0
Length, mm
Root	1265 ± 60.1	0
Stem	449 ± 24.69	0
Seedling fresh mass, mg	1525.72 ± 507.63	0
Seedling dry mass, mg	163.57 ± 34.78	0
Water content, %	7.0ˆa ± 0.08	4.8^b^ ± 0.41
Electroconductivity, mS g^-1^	810ˆa ± 41.04	1889.67^b^ ± 44.86

### Protein Content

The protein fractions content in seeds stored at -14°C was 14, 4, and 69 mg g^-1^ for γ, δ, and β conglutin, respectively. On the other hand, in the seeds stored at +20°C it was 15.5, 3, and 65 mg g^-1^ (**Table 2**).

**Table 2:** The number of protein spots in gels and the average protein fractions (mg g^-1^) extracted from lupin seeds stored at -14 and + 20°C for 26 years at different temperatures (data are expressed as the mean of 3–5 independent repeats ± SD, means in each column followed by the same letter are not significantly different *p* ≤ 0.01).

	Storage temperature	
	-14°C	+20°C
Protein content, mg g^-1^
γ	14ˆa ± 0.6	15.5ˆa ± 0.8
δ	4ˆa ± 0.3	3ˆa ± 0.5
β	69ˆa ± 0.2	65ˆa ± 0.3
Number of spots
γ and δ	310ˆa ± 2	228ˆb ± 3
β 12.5%	172ˆa ± 5.03	153ˆb ± 8.5
β 10%	223ˆa ± 5.51	141ˆb ± 5.51

**FIGURE 1:**
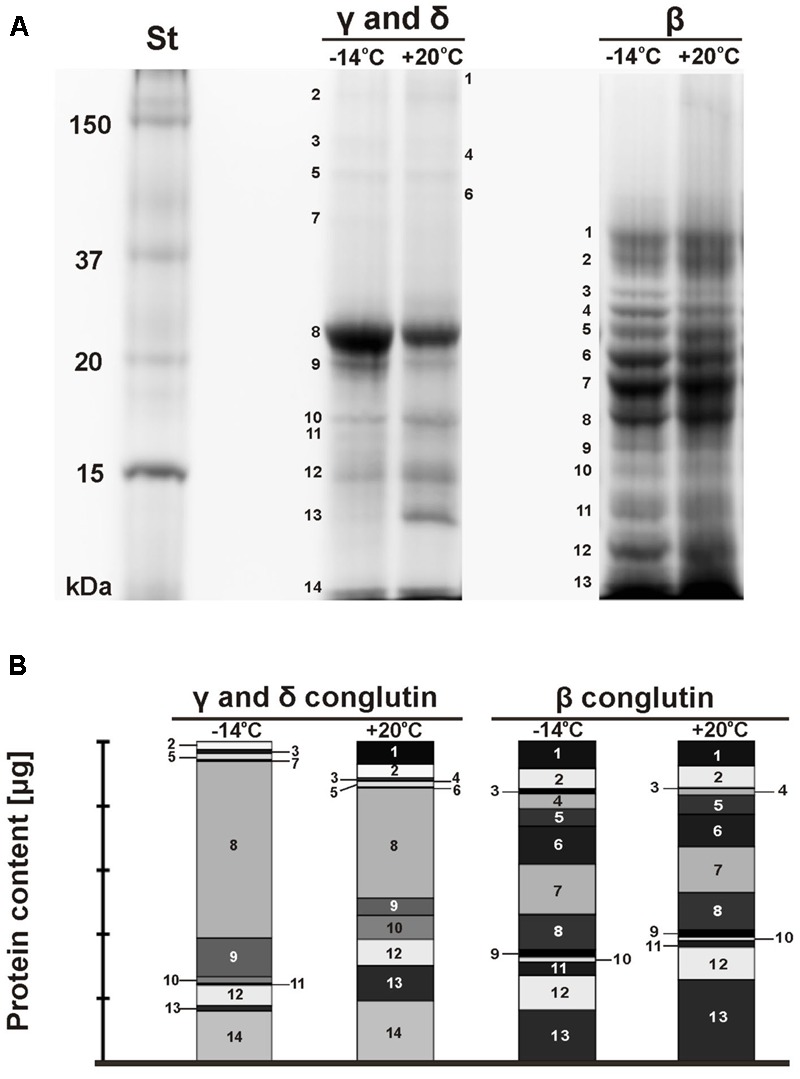
SDS-PAGE electrophorograms of lupin proteins. The seed were stored at different temperatures (**A**: St-protein standards, –14°C, +20°C – storage temperature, γ, δ, and β conglutins; **B**: γ, δ, and β conglutin contents) during the 26–years period. On the original gel the separation lines for γ and δ conglutins on one hand and β conglutins on the other hand were directly adjacent. They were shifted apart in this figure for clarity.

Electrophorograms of γ, δ, and β conglutin fractions obtained from seeds stored at -14°C and +20°C were clearly different (**Figure 1A**). The differences mainly related to the intensity of particular bands, but also to the number of bands in particular fractions. The paths corresponding to γ and δ conglutins, obtained from seeds stored at -14°C and at +20°C, were characterized by the presence of several intense bands of polypeptides with weights from 23.0 to 10.3 kDa. Polypeptide bands with molecular weights of 22.4 (band 8) and 19.8 kDa (band 9) exhibited almost two times higher expression in the seeds stored at -14°C (**Figure 1B**). On the other hand, polypeptides with weights of 17.1 (band 10) and 13.1 kDa (band 13) exhibited the maximum expression in the seeds stored at +20°C, and the polypeptide with a weight of 17.1 kDa was two times higher (**Figure 1B**). The fading of the band with a weight of 16.4 kDa (band 11) was observed in the seeds stored at +20°C.

The set of polypeptide bands corresponding to β conglutins in both temperature variants was identical (**Figure 1A**). The polypeptide bands ranged from 41.0 to 10.4 kDa. The polypeptides with weights of 26.9 (band 4) and 13.5 kDa (band 11) in the seeds stored at -14°C was two times higher than in the seeds stored at a room temperature (**Figure 1B**). However, the expression level of the polypeptide band with a weight of 30 kDa (band 3) was six times higher. The maximum expression in the seeds stored at +20°C was demonstrated by one polypeptide band with a weight of 10.4 kDa (band 13). In the analyzed seeds, the presence of polypeptide subunits corresponding to γ and δ conglutins was also found. This confirms that it is often impossible to obtain a uniform function.

### Two-Dimensional Gel Electrophoresis and Protein Identification

**FIGURE 2:**
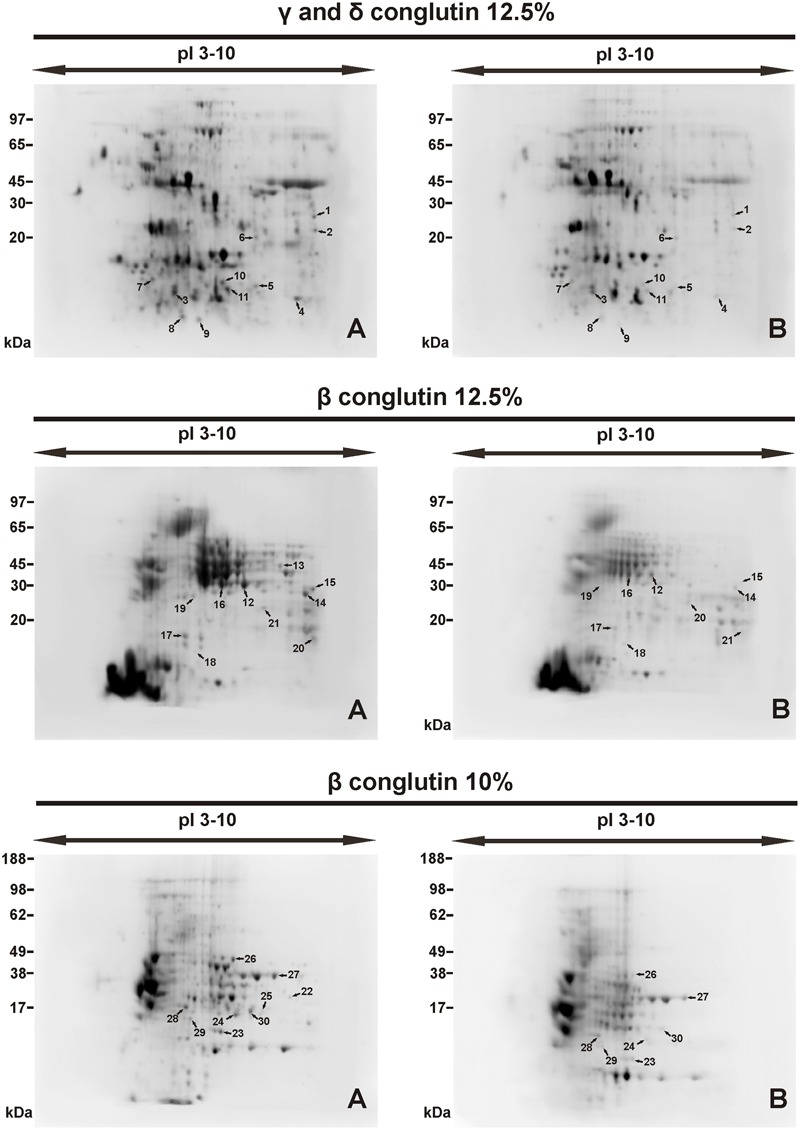
Protein spots on 2D-electrophorograms of seed proteins. Spot location **(A)** seed stored at -14°C, **(B)** seed stored +20°C. Conglutin separation was conducted at pI 3–10. Number of spots 1, 2....30 – see in **Table 3**.

**FIGURE 3:**
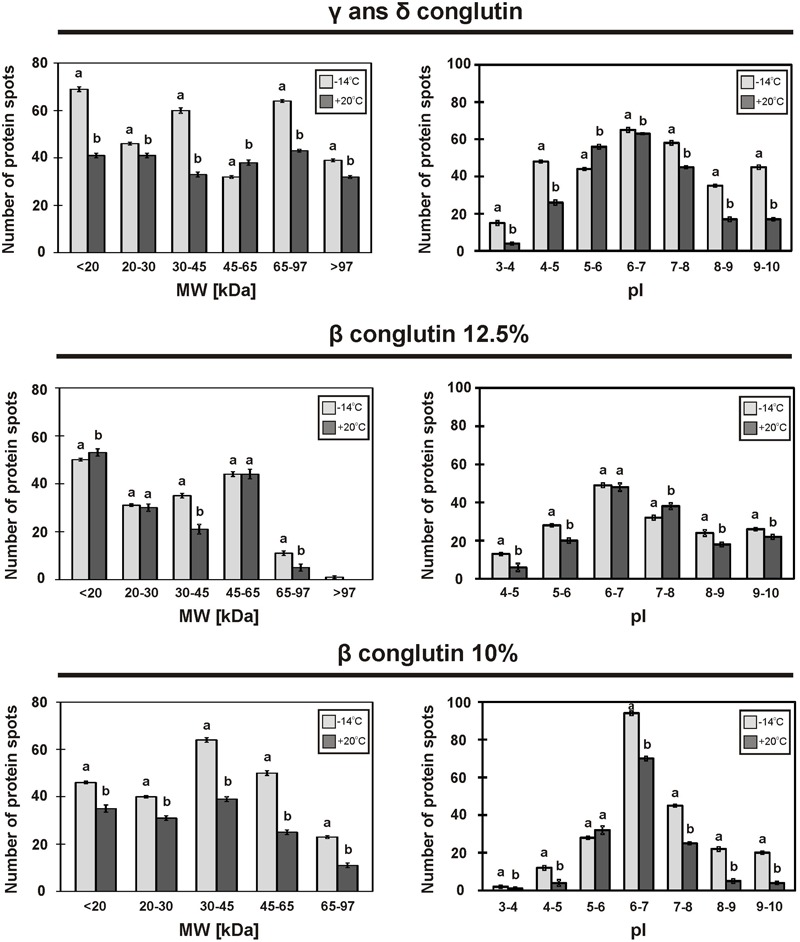
Molecular weight distribution of proteins fractions on 2D-electrophorograms, seed stored at -14°C (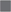) and +20°C (■). Means with the same letter are not significantly different from each other (*p* ≤ 0.01).

**FIGURE 4:**
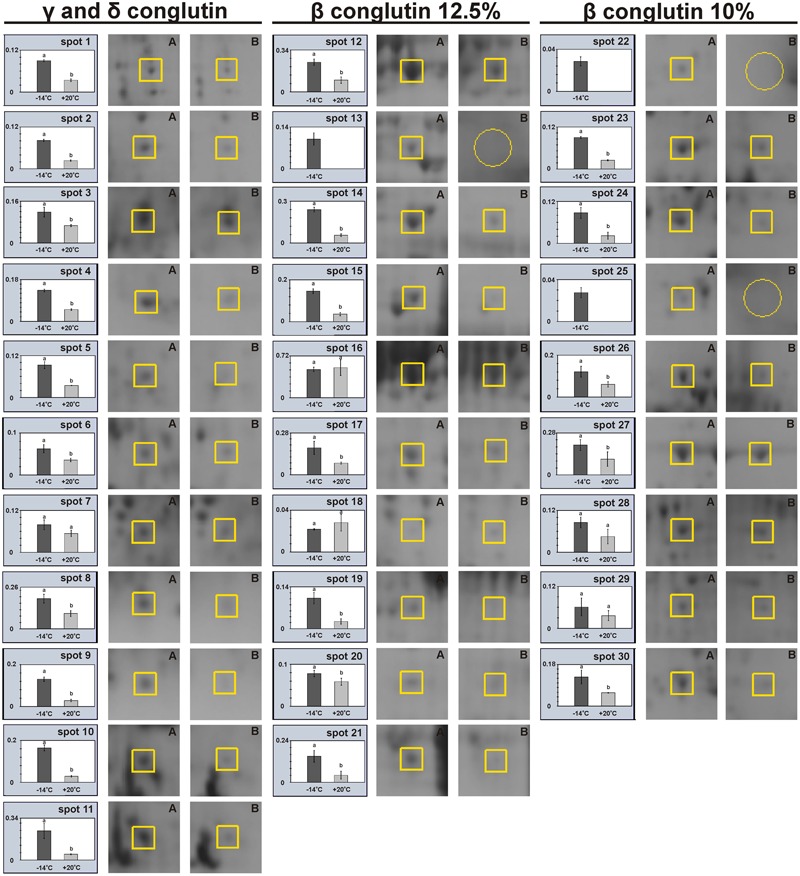
Relative levels of protein expression on 2D-electrophorograms, **(A)** seed stored at -14°C (■) and **(B)** seed stored at +20°C (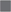). An open circle on a number line means no protein exists.

The general patterns of the tested protein fractions are similar in the seeds stored at -14°C and at +20°C (**Figure 2**). However, the number of spots and their intensity differs between the seeds stored under different conditions (**Figures 3, 4**).

#### γ and δ Conglutins

2D-PAGE images of γ and δ conglutins obtained from the seeds stored at -14°C and +20°C are presented in **Figure 2**. Over the entire range of molecular weights, an average of 310 protein spots were observed on the maps generated for the seeds stored at -14°C. On the other hand, on the maps obtained for seeds stored at room temperature, an average of 228 spots were observed (**Table 2**). Most polypeptide subunits had pI ranging from 4.5 to 7 and a molecular weight of 10–97 kDa. The greatest diversity of polypeptides between the analyzed variants was observed within the molecular weight range of 20–45 kDa (-14°C: 175, +20°C: 115 protein spots), and within the range of 65–97 kDa (-14°C: 103, +20°C: 75 protein spots) (**Figure 3**). On a map generated for the seeds stored at +20°C, a clear decline in basic polypeptides (8–10 pI) (**Figure 3**) was observed. Eleven proteins were analyzed by mass spectrometry. The results of identification are shown in **Table 3**. One protein (spot 7) was similar to δ conglutin. Eight proteins (spots 2, 4, 5, 6, 8, 9, 10, 11) were identified as γ conglutins. A single protein was a glyceraldehydes-3-phosphate dehydrogenase (GAPDH) (spot 3). The content of the identified proteins in seeds differed significantly between the tested variants. In the seeds stored at -14°C all analyzed proteins occurred at the highest levels (**Figure 4**).

**Table 3:** Proteins in white lupin seeds indentified by LC-MS-MS/MS analyses.

Spot	Protein identification	Protein ID	MW^∗^		pI^∗^		Score	Sequence coverage %	Role
			Th	Exp	Th	Exp			
1	Conglutin beta 1	gi| 122220821	61.99	26.00	6.08	9.48	562	17	Storage
2	Conglutin gamma	gi| 11191819	49.87	22.50	8.39	9.48	394	11	Storage
3	GAPDH	gi| 62816190	32.28	9.68	6.80	5.75	867	30	Carbohydrate and energy metabolism
4	Conglutin gamma	gi| 11191819	49.87	9.40	8.39	8.84	544	15	Storage
5	Conglutin gamma	gi| 11191819	49.87	11.87	8.39	7.80	1184	32	Storage
6	Conglutin gamma	gi| 11191819	49.87	20.50	8.39	7.76	627	21	Storage
7	Conglutin delta seed storage protein precursor	gi| 80221495	17.64	15.50	5.47	5.20	462	48	Storage
8	Conglutin gamma	gi| 11191819	49.87	6.88	8.39	6.00	312	12	Storage
9	Conglutin gamma	gi| 11191819	49.87	6.25	8.39	6.34	302	13	Storage
10	Conglutin gamma	gi| 11191819	49.87	11.90	8.39	7.00	891	22	Storage
11	Conglutin gamma	gi| 11191819	49.87	11.25	8.39	7.12	1484	33	Storage
12	Conglutin beta 1	gi| 122220821	61.99	28.75	6.08	5.51	2756	60	Storage
13	Conglutin beta 1	gi| 122220821	61.99	41.30	6.08	8.50	2391	56	Storage
14	Conglutin beta 1	gi| 122220821	61.99	25.85	6.08	9.20	2074	48	Storage
15	Conglutin beta 1	gi| 122220821	61.99	27.50	6.08	9.42	1995	50	Storage
16	Conglutin beta 1	gi| 122220821	61.99	30.00	6.08	7.00	2923	58	Storage
17	Conglutin beta 2	gi| 75121065	62.09	16.88	6.43	6.99	1601	40	Storage
18	Conglutin beta 1	gi| 122220821	61.99	14.37	6.08	6.31	1579	39	Storage
19	Conglutin beta 1	gi| 122220821	61.99	25.00	6.08	6.29	2434	61	Storage
20	Conglutin beta 1	gi| 122220821	61.99	22.50	6.08	8.12	1627	42	Storage
21	Conglutin beta 2	gi| 75121065	62.09	16.25	6.43	9.50	1588	39	Storage
22	Conglutin beta 1	gi| 122220821	61.99	22.50	6.08	8.60	1474	34	Storage
23	Conglutin beta 1	gi| 122220821	61.99	15.25	6.08	6.85	1127	27	Storage
24	Conglutin beta 1	gi| 122220821	61.99	18.65	6.08	7.25	1417	32	Storage
25	Conglutin beta 1	gi| 122220821	61.99	19.68	6.08	8.00	1965	46	Storage
26	Conglutin beta 2	gi| 75121065	62.09	41.25	6.43	7.20	1695	44	Storage
27	GAPDH	gi| 62816190	32.28	27.50	6.80	8.28	1209	47	Carbohydrate and energy metabolism
28	Conglutin beta 1	gi| 122220821	61.99	20.00	6.08	6.04	1501	38	Storage
29	Conglutin beta 1	gi| 122220821	61.99	17.50	6.08	6.10	1483	38	Storage
30	Conglutin beta 1	gi| 122220821	61.99	19.37	6.08	7.60	1905	46	Storage

*^∗^MW, molecular weight; pI, isoelectric point; Th, theoretical; Exp, experimental.*

#### β Conglutin in 12.5% Polyacrylamide Gel

A map generated for the seeds stored at -14°C contained an average of 172 spots, while for the seeds stored at +20°C, it contained 153 spots (**Figure 2** and **Table 2**). Most polypeptides corresponding to β conglutins fell within the ranges of 10–65 kDa and 5–8 pI. In both variants, electrophoregrams exhibited great similarity. However, they differed in the intensity of protein spots, particularly in the region including 30–65 kDa and 5–7.5 pI (**Figure 2**). Moreover, in the seeds stored at a room temperature, a decline in polypeptides with a molecular weight higher than 95 kDa was observed (**Figure 3**). Ten proteins were analyzed by mass spectrometry (**Table 3**). All of them were similar to β conglutin. Only two proteins (spots 16, 18) of all the identified proteins occurred at a higher level in seeds stored at room temperature. Moreover, in the seeds stored at a room temperature, a disappearance of a β conglutin subunit from site 13 was noted (**Figures 2, 4**).

#### β Conglutin in 10% Polyacrylamide Gel

The fraction obtained from seeds stored at -14°C was characterized by the average presence of 223 protein spots, while that from the room temperature seeds was characterized by the presence of 141 spots (**Figure 2** and **Table 2**). Most subunits corresponding to β conglutins fell within the ranges of 10–65 kDa and 5–8 pI. The region with the greatest diversity between the tested variants included a group of polypeptides with a molecular weight of 20–65 kDa (-14°C: 145, +20°C: 95 spots) (**Figure 3**). Nine proteins of that fraction were identified (**Table 3**). Eight proteins (spots: 22, 23, 24, 25, 26, 28, 29, 30) were similar to β conglutin, while the others were similar to glyceraldehydes-3-phosphate dehydrogenase (GAPDH) (spot 27). Seeds stored at -14°C were characterized by considerably higher quantities of studied proteins (**Figure 4**). In 2D-PAGE of proteins from seeds stored at room temperature, the disappearance of spots 22 and 25 of β conglutin subunits from was observed (**Figures 2, 4**).

## Discussion

Storage conditions such as the temperature and humidity affect the viability of seeds (Parrish and Leopold, 1978; Jatoi et al., 2001; Mohammadi et al., 2011). White lupin seeds, despite the long period of storage (26 years) under the conditions of lowered temperature, retained viability at a level of 86.3% (**Table 1**). This observation corroborates previous studies suggesting that low seed storage temperature has a positive effect on seed storability (Cortelazzo et al., 2005; Sastry et al., 2007; Balešević-Tubić et al., 2010; Pradhan and Badola, 2012). Seeds of lupin are considered orthodox, i.e., they tolerate well drying to a water content of 5–10% dry weight (Piotrowicz-Cieślak et al., 2008). Therefore, they retain the ability to germinate for a long time. It has been established that orthodox seeds best retain their viability when stored at a temperature below 0°C and RH of approx. 5–8% (Sastry et al., 2007; Shaban, 2013). Walters et al. (2005) reported that the viability of the seeds of narrow-leafed lupin (*L. angustifolius* L.) dropped by 50% after 41 years of storage at -18°C. Piotrowicz-Cieślak et al. (2008) established that the viability of yellow lupin seeds after 20 years of storage at -14 and 0°C was similar and remained at a level of 99 and 96%, while the viability of the seeds stored at +20°C dropped to only 2%. The results obtained by Piotrowicz-Cieślak et al. (2008) confirm that a temperature of 0°C was sufficient for maintaining the viability of lupin seeds at a high level. According to Elias and Copeland (1994), a temperature of 5°C contributes to the slowing down of metabolic processes that cause deterioration of seed material. Lee et al. (2013), while comparing the viability of seeds of selected species of the *Fabaceae* family, noted an approx. 15% drop in the viability of pea (*Pisum sativum* L.) and soybean (*Glycine max* Merr.) seeds after 10 years of storage at +4°C. It was found in the same study that the most durable seeds are those of *Arachis hypogaea, Vigna angularis* var. *angularis*, and *Phaseolus vulgaris*, whose viability decreased by only 5%. On the other hand, in a study by Walters et al. (2005), the best storability was exhibited by pea seeds. After long-term storage at -18°C, a drop in the viability by 25% was noted in pea seeds (after 64 years of storage). As regards pea seeds, an above-zero storage temperature is not sufficient for them to retain their maximum durability.

Maintaining the RH of the air and water content of seeds at a low level is also significant for retaining a high quality of seed material (Priestley, 1986; Ratajczak et al., 2015). However, according to Mira et al. (2015), during storage at a temperature lower than +30°C, a reduction in the amount of water in seeds has no effect on their storability. The water content of the seeds at the beginning of the experiment was 8.8%, while after 26 years of storage at -14 and +20°C it was, respectively, 7.0 and 4.8%. Ellis and Hong (2006) concluded in their study that changes in water content at a level of 2–15% during the storage of seeds under the conditions of a lowered temperature had no effect on the longevity of seeds of plants of the *Fabaceae* family. Similar conclusions were reached by Pérez-García et al. (2007) in a study on the seed longevity of plants of the *Brassicaceae* family. Given these results, in our experiment we excluded the RH of the air and water content of seeds as a factor affecting the durability of white lupin seeds.

According to Tabe et al. (2002) and Shaban (2013), the quantity and quality of stored proteins may vary depending on the impact of various factors. In seeds stored at room temperature, the contents of γ, δ, and β conglutins decreased. A similar relationship was observed by Cortelazzo et al. (2005) in the seeds of French beans (*P. vulgaris* L.), Piotrowicz-Cieślak et al. (2008) in yellow lupin and Mbofung et al. (2013) in soybean. A direct relationship between seed viability and vigor and seed storage proteins was confirmed in a study by Machado et al. (2001), Mbofung et al. (2013), and Sathish et al. (2015). The changes in structural proteins of non-germinating seeds of white lupin are indicated by a reduction in the intensity of protein spots and the fading of the band with a molecular weight of 16.4 kDa (band 11, fractions γ and δ) (**Figures 1A,B**). A similar fading of bands in aging bean seeds was observed by Machado et al. (2001). Along with a gradual loss of seed viability, the authors observed a gradual disappearance of polypeptide bands in the seed protein profile. According to Rajjou et al. (2008) and Sathish et al. (2015), the fading of polypeptide bands indicates post-translational modifications and protein degradation occurring during seed storage. According to Gao et al. (2016), the content of storage proteins is the main determinant of seed longevity. This statement is supported by the fact that the biggest differences between seeds with high and low durability are seen in their storage proteins. Furthermore, it was shown that the degradation of storage proteins during seed storage leads to disturbances in the supply of amino acids necessary for the synthesis of new proteins required during germination and seedling formation (Sathish et al., 2015).

The application of two-dimensional electrophoresis for study of seed proteins is a challenge. A 2D-electrophorogram generated for seed proteins is often dominated by the main storage proteins (particularly β conglutins) which mask other fractions and functional categories. Therefore, in our experiment we decided to carry out a preliminary fractionation of white lupin seed proteins before separating them by electrophoresis. In a study of narrow-leafed lupin seed proteome, published by Islam et al. (2012), the 2D-electrophorogram comprised mostly of polypeptides similar to β conglutin. On the other hand, in gels with yellow lupin seed proteins 205 β conglutin spots were found among a total of 341 seed protein spots (Ogura et al., 2014). The performance of preliminary protein fractionation proceeding the 2D electrophoresis in our experiments enabled us to identify proteins that change during seed storage.

The identified proteins (**Table 3** and **Figure 2**) were storage proteins: β conglutins (belonging to 7S globulins or vicilin-like proteins), γ conglutins (belonging to 7S globulins or vicilin-like proteins), δ conglutins (belonging to 2S albumins), and proteins responsible for bioenergetic metabolism, i.e., glyceraldehydes-3-phosphate dehydrogenase (GAPDH). In our experiment, the amount of all polypeptides identified as γ and δ conglutins decreased in the seeds stored at room temperature (**Figures 2, 4**). As regards the polypeptides identified as β conglutins, nineteen occurred in higher amounts in seeds stored at -14° C, while two (spots 16 and 18) dominated in seeds stored at 20°C. Changes in contents of vicilin-like proteins were demonstrated in aging seeds of maize (*Zea mays* L.) (Xin et al., 2011) and rice (*Oryza sativa* L.) (Gao et al., 2016). Xin et al. (2011), while observing the rapid disappearance of vicilin-like proteins, concluded that the level of these proteins is correlated with the rate of germination and seedling growth. It is suggested that vicilin-like proteins, in addition to the role of nutrient reservoir, may serve additional functions. The disappearance of 2S albumins was observed in the seeds of *Arabidopsis* (Nguyen et al., 2015). Mature seeds of *Arabidopsis*, devoid of this fraction, were characterized by a reduced total protein content and were more susceptible to aging. Stability of γ conglutin during seed germination and its resistance to proteolysis make the storage function of this protein rather doubtful. It is exuded from seeds under stress conditions and during germination (Scarafoni et al., 2013). Due to the high homology of γ conglutin to glycoside hydrolase, it was suggested that this protein may participate in cell defense reactions. It was demonstrated by Scarafoni et al. (2010) γ conglutin from white lupin does not inhibit the cell wall degrading enzymes. Ribeiro et al. (2014) classify γ, β conglutins and albumins as family II of legume lectins. Lectins bind carbohydrates and are involved in plant defense from insects and pathogens, interactions with symbionts and regulation of cell adhesion. Moreover, these proteins probably participate in the signal transduction pathways, induce hypersensitivity reaction and strengthen the cell wall and cell membrane (Ribeiro et al., 2014; De Oliveira Dias et al., 2015). Jimenez-Lopez et al. (2016) have found that the β conglutins facilitate the oxidative burst, while at the same time they prevent its extinguishing and induction of apoptosis. The protective function of δ conglutins is also suggested by their similarity to the alpha-amylase inhibitor of cereal plants (Duranti et al., 2008). Nguyen et al. (2015) have shown that the decay of seed albumins resulted in decreased longevity of seeds and their greater sensitivity to oxidation. These authors suggested that albumins play a buffering role and provide protection from oxidative stress for other proteins necessary at the later stages of seed germination and seedling growth. Our experiments confirmed that γ, δ and β conglutins deteriorate parallel to the deterioration of seed vigor and longevity. This was demonstrated by one- and two-dimensional electrophoresis of the extracted protein fractions.

## Author Contributions

MD performed molecular experiments, AP-C supervised the research design and wrote the manuscript. All authors discussed the results and commented on the manuscript.

## Conflict of Interest Statement

The authors declare that the research was conducted in the absence of any commercial or financial relationships that could be construed as a potential conflict of interest.
